# New National Air-Kerma-Strength Standards for ^125^I and ^103^Pd Brachytherapy Seeds

**DOI:** 10.6028/jres.108.030

**Published:** 2003-10-01

**Authors:** Stephen M. Seltzer, Paul J. Lamperti, Robert Loevinger, Michael G. Mitch, James T. Weaver, Bert M. Coursey

**Affiliations:** National Institute of Standards and Technology, Gaithersburg, MD 20899-0001

**Keywords:** air kerma, brachytherapy seed source, exposure, free-air chamber, ^125^I, national measurement standard, ^103^Pd, x rays

## Abstract

The new U.S. measurement standard for the air-kerma strength from low-energy photon-emitting brachytherapy seed sources is formally described in detail. This instrument-based standard was implemented on 1 January 1999, with its salient features and the implications of differences with the previous standard given only through a series of informal communications. The Wide-Angle Free-Air Chamber (WAFAC) is specially designed to realize air kerma from a single-seed source emitting photons with energies up to about 40 keV, and is now used to measure the wide variety of seeds used in prostate-cancer therapy that has appeared in the last few years. For the two ^125^I seed models that have been subject to both the old and new standards, the new standard reduces the air-kerma strength by 10.3 %. This change is mainly due to the removal of the influence on the measurement of the Ti K x rays produced in the source encapsulation, a component with no clinical significance.

## 1. Introduction

The National Institute of Standards and Technology (NIST), formerly the National Bureau of Standards (NBS), maintains the U.S. primary standards for air kerma (formerly exposure) for x rays generated at potentials in the range from 10 kVp to 300 kVp using a series of free-air ionization chambers. Three parallel-plate chambers were constructed more than 30 years ago, characterized to provide x-ray air-kerma standardization within this energy range, and are used almost exclusively in the W-anode x-ray calibration facilities at NIST. These free-air chambers are identified as the Lamperti chamber [1] for 10 kVp to 20 kVp, the Ritz chamber [2,3] for 20 kVp to 100 kVp, and the Wycoff-Attix chamber [4] for 50 kVp to 300 kVp. More recently, NIST added a new cylindrical free-air ionization chamber of an Attix design [5] to provide air-kerma standardization for mammography x-ray beams from both Mo and Rh anodes operated in the 23 kVp to 40 kVp range [6].

Brachytherapy sources are small, encapsulated radioactive sources used for interstitial, intracavity, intraluminary or applicator radiation therapy (*brachy* is borrowed from the Greek, meaning “short,” to describe the small or contact distances involved in such therapy). Primary standards for the air kerma from photon-emitting radionuclides have been developed by the NBS/NIST as well as by other national metrology institutes. The NBS standardization of ^137^Cs sources is based on the use of appropriate Bragg-Gray cavity chambers, as described by Loftus [7]; a similar standardization of ^60^Co and ^137^Cs gamma-ray beams is detailed by Loftus and Weaver [8]. The use of Bragg-Gray cavity chambers formed the basis for the earlier NBS standardization of ^192^Ir sources by Loftus [9]. A change in the analyses of the results from these Bragg-Gray cavity-chamber standards is detailed by Seltzer and Bergstrom [10].

To provide similar traceability to NBS exposure standards for the case of the low-energy photon-emitting ^125^I brachytherapy seeds then available, Loftus [11] performed measurements with the national primary x-ray standard Ritz free-air chamber, and transferred the results to a spherical aluminum re-entrant ionization chamber which then served as the secondary standard for routine calibrations. By the early 1990s, deficiencies in the standard and the need to calibrate seeds of newer design, particularly those incorporating ^103^Pd instead of ^125^I, prompted NIST to develop a new standard for these brachytherapy seeds. The new standard was formally introduced on 1 January 1999, and numerous calibrations based on that standard have been performed for the still-growing number of new seed designs. The purpose of this report is to fully document the new NIST air-kerma-strength standard for these ^125^I and ^103^Pd brachytherapy seeds that emit photons with energies up to about 40 keV.

## 2. Relevant Quantities

The quantity kerma (an acronym for kinetic energy released per unit mass), *K*, characterizes a beam of photons or neutrons in terms of the energy transferred to any material. Kerma is defined [12] as the quotient of d*E*_tr_ by d*m*, where d*E*_tr_ is the sum of the initial kinetic energies of all the charged particles liberated by uncharged particles (in our case, photons) in a mass d*m* of material. Thus,

$$K=\frac{dE_{\text{tr}}}{dm}.$$ (1)

The SI unit of kerma is the gray (Gy), which is equal to one joule per kilogram (J kg^−1^). Kerma rate, *K*, is the quotient of d*K* by d*t*, where d*K* is the increment of kerma in the time interval d*t*. Our interest is in air kerma, *K*_air_, where d*m* is the mass of air.

The exposure, *X*, is defined [12] as the quotient of d*Q* by d*m*, where d*Q* is the absolute value of the total charge of the ions of one sign produced in air when all the electrons and positrons liberated or created by photons in air of mass d*m* are completely stopped in air. Thus,

$$X=\frac{dQ}{dm}.$$ (2)

The SI unit of exposure is C kg^−1^; however, the older unit of Roentgen (R) is still used by some, where 1 R = 2.58 × 10^−4^ C kg^−1^. The quantities exposure and air kerma can be related through use of the mean energy per unit charge, *W*/e, where *W* is the mean energy expended in air per ion pair formed when the initial kinetic energy of a charged particle is completely dissipated in the air, and *e* is the elementary charge. Then

$$K_{\text{air}}=X\cdot (W/e)/(1-\bar{g}).$$ (3)

The quantity *g* is the fraction of the kinetic energy of electrons (and positrons) liberated by the photons that is lost in radiative processes (mainly bremsstrahlung) in air. In Eq. (3), $\bar{g}$ is the mean value of *g* averaged over the distribution of the air kerma with respect to electron energy. For the low-energy photons (< 40 keV) emitted by ^125^I and ^103^Pd seeds, $\bar{g}$ is very small (< 0.00065) and is taken to be zero. The value of *W*/*e* for dry air currently adopted by the international measurement system is (33.97 ± 0.05) J/C [13], where the uncertainty pertains to one standard deviation.

Small brachytherapy sources usually have an external shape of that of a right circular cylinder, perhaps with rounded end-caps. As recommended by the American Association of Physicists in Medicine (AAPM), the air-kerma strength, *S*_k_, is defined [14] for these sources as the product of the air-kerma rate at a point in free space (vacuo) located in the transverse bisecting plane at a distance *d* from the center (i.e., cylindrical axis) of the seed, and the square of the distance *d*. Thus,

$$S_{k}={\dot{K}}_{\text{air}}(d)\cdot d^{2}.$$ (4)

The calibration distance *d* should be large enough that the source may be treated as a mathematical point^1^. SI units of air-kerma strength are Gy m^2^ s^−1^; units more appropriate for sources of interest here (in which typical values would be roughly of the order of unity) are µGy m^2^ h^−1^, given the special symbol U by the AAPM. The quantity air-kerma strength is used in North America; the corresponding quantity used internationally is the reference air-kerma rate in vacuo, at a specified reference calibration distance, with units µGy h^−1^. The reference calibration distance is usually specified as 1 m, in which case air-kerma strength and reference air-kerma rate would have the same numerical value, although formally with different units. As all kerma and kerma rates will be that for air in the remainder of this report, further use of the subscript *air* will be dropped for simplicity.

A somewhat related quantity is the air-kerma-rate constant, *Γ_δ_*, of a photon-emitting radionuclide, defined as

$$\Gamma_{\delta }=\frac{d^{2}\cdot {\dot{K}}_{\delta }}{A},$$ (5)

where ${\dot{K}}_{\delta }$ is the air-kerma rate due to photons of energy greater than *δ*, at a distance *d* in vacuo from a point source of the nuclide having an activity *A*. The units of *Γ_δ_* are Gy m^2^ s^−1^ Bq^−1^. The quantity *AΓ_δ_* then is the analog of air-kerma strength, but for a true point source. The relationship also is the basis for a definition of the *apparent* activity of a source: *A*_app_ = *S*_k_/*Γ_δ_*, where *S*_k_ is for the real, encapsulated source, but *Γ_δ_* is for a true point source of the same nuclide.

Kerma and exposure can be evaluated in terms of the photon fluence and interaction coefficients. The fluence, *Φ*, is the quotient of d*N* by d*a*, where d*N* is the number of particles incident on a sphere of cross-sectional area d*a*. The distribution of fluence with respect to energy is given by *Φ_E_* = d*Φ*/d*E*. The photon mass energy-transfer coefficient, *µ*_tr_/*ρ*, is the quotient of d*E*_tr_/*E* by *ρ*d*ℓ*, where d*E*_tr_/*E* is the fraction of the incident energy that is transferred to kinetic energy of charged particles by interactions in traversing a distance d*ℓ* in a material of density *ρ*. Then kerma is given by

$$K=\int \Phi_{E}E\frac{\mu_{\text{tr}}}{\rho }dE.$$ (6)

and exposure is given by

$$X=\frac{e}{W}\int \Phi_{E}E\frac{\mu_{\text{tr}}}{\rho }(1-g)dE.$$ (7)

The quantity $\frac{\mu_{\text{tr}}}{\rho }(1-g)$ is the photon mass energy-*ρ* absorption coefficient, *µ*_en_/*ρ*. As noted previously, however, there is no practical difference between *µ*_tr_ and *µ*_en_ for photons with energies of interest here, as *g* is negligible. Note that these fluence-based quantities are expressed at a point. Exposure and air kerma can be expressed at a point in a material other than air, such as water or a vacuum.

For therapy applications, the quantity of most direct interest for these seeds is the absorbed-dose rate at a reference point in tissue or in water. However, a primary measurement standard should be directly realizable. The absorbed-dose rate at a point in water cannot be measured absolutely for these sources, but it is proportional to the air-kerma rate for the source, which can be measured absolutely through use of a free-air chamber (FAC).

## 3. Principles of a Free-Air Chamber

A few details on the conceptual application of a free-air chamber to these measurements are useful. In a free-air chamber, a circular aperture at the point of measurement admits a beam of photons that travel free in air through a well-defined volume in which the charge generated by the interaction of the photons with the air is collected. The collecting volume is usually large enough in the direction perpendicular to the beam axis so that the radial dimension of the collection volume captures the first interaction of the photons and the subsequent ionization produced by the secondary electrons. The length (along the beam axis) of the collecting volume is large enough to produce a quantity of charge sufficient for an accurate measurement. The measured charge is corrected for a number of effects, most obviously including the attenuation of the photon beam in the air within the FAC, the charge produced in the FAC by scattered photons, and the loss of charge by secondary electrons absorbed in material other than the collecting air volume. These corrections are required to relate the charge measured in the large volume of the FAC to the differential amount of charge d*Q*, per differential amount of mass of air d*m*, needed to realize the definitions of air kerma and exposure, given by Eqs. (1) and (2).

**Fig. 1:**
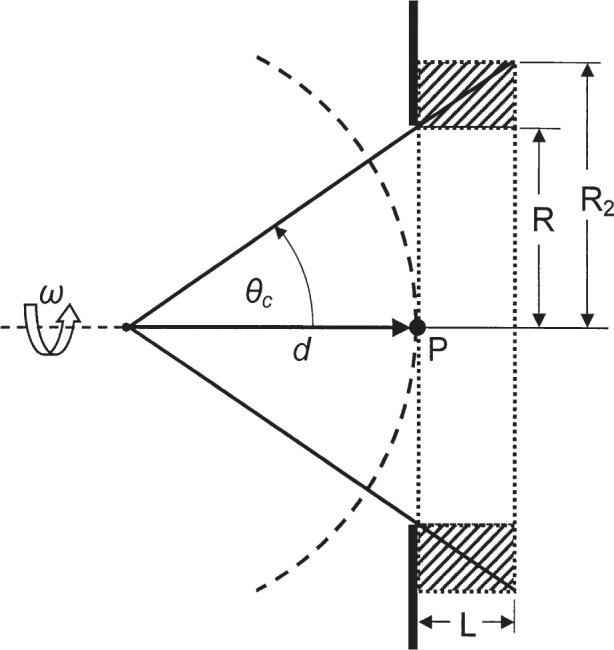
Schematic diagram of a point-isotropic source whose emitted “rays” are admitted into the detector by a plane aperture of radius *R* at distance *d* from the source. The detected tracks form a cone of half-angle *θ*_c_ that expands to a radius *R*_2_ over the counting length *L*. The desired quantity is the track fluence at point P.

Equivalently, Eqs. (6) and (7) can be used for the definitions of exposure and air kerma, and one then needs only to relate a measurement of the average fluence measured for a volume to the value at a point. Consider the schematic in Fig. 1. A point-isotropic source emits photons in vacuum. There is no loss in generality if the photons are considered here to be monoenergetic. An aperture of radius *R* is placed in the plane at point P, followed by the cylindrical measuring volume of length *L*. The beam subtends a measuring volume, or total-track detector, of radius *R*_2_ > *R*. It can be shown [15] that the average fluence in a volume *V* is

$$\bar{\Phi }=y/V,$$ (8)

where *y* is the total tracklength in that volume. Referring to Fig. 1, *R*_2_ = *R*·(1 + *L*/*d*), and the circular aperture defines a conical beam with half-angle *θ*_c_ such that cos*θ*_c_ = [1 + (*R*/*d*)^2^]^−1/2^. The total tracklength in the measuring volume is then simply

$$y=\int_{0}^{2\pi }d\omega \int_{\cos\theta_{c}}^{1}d(\cos\theta )f(\cos\theta )\frac{L}{\cos\theta },$$ (9)

where *ω* is the azimuthal angle and *f* is the angular distribution of the emitted radiation. For the point isotropic source, *f* = 1/4π, and the integral over *ω* is simply 2π. Then

$$y=\frac{2\pi }{4\pi }L\int_{\cos\theta_{c}}^{1}\frac{d\cos\theta }{\cos\theta }=\frac{L}{2}\ln(1/\cos\theta_{c})=\frac{L}{4}\ln[1+{\left(R/d\right)}^{2}].$$ (10)

The volume in our total-track detector defined by the conical beam is

$$V=\pi R_{2}^{2}L=\pi R^{2}{\left(1+L/d\right)}^{2}L,$$ (11)

so that the average fluence in the detector of length *L* is then

$${\bar{\Phi }}_{R,L}(d)=\frac{y}{V}=\frac{\ln[1+{\left(R/d\right)}^{2}]}{4\pi R^{2}{\left(1+L/d\right)}^{2}}.$$ (12)

In the limit *L* → 0 (the total-track detector squeezed down to a plane detector),

$${\bar{\Phi }}_{R,0}(d)=\frac{\ln[1+{\left(R/d\right)}^{2}]}{4\pi R^{2}}=\frac{1}{4\pi d^{2}}{\left(\frac{d}{R}\right)}^{2}\ln[1+{\left(R/d\right)}^{2}].$$ (13)

From first principles, the fluence at the point P at distance *d* is

$$\Phi (d)=\frac{1}{4\pi d^{2}},$$ (14)

so that the quantity

$$\frac{\Phi (d)}{{\bar{\Phi }}_{R,0}(d)}=\frac{{\left(R/d\right)}^{2}}{\ln[1+{\left(R/d\right)}^{2}]}$$ (15)

is simply the correction factor to relate the fluence averaged over the planar aperture of radius *R* to the fluence at the point at distance *d*.

The correction factor that relates ${\bar{\Phi }}_{R,L}(d)$to ${\bar{\Phi }}_{R,0}(d)$ is the remaining factor in Eq. (12), i.e.,

$$\frac{{\bar{\Phi }}_{R,0}(d)}{{\bar{\Phi }}_{R,L}(d)}={\left(1+L/d\right)}^{2}.$$ (16)

Applying this correction factor, to refer the measured fluence averaged over the volume to the fluence averaged over the planar aperture, is equivalent to simply replacing the volume *V* in Eq. (11) by an effective volume given by

$$V_{\text{eff}}=V/{\left(1+L/d\right)}^{2}=\pi R^{2}L.$$ (17)

That is, the effective volume is the product of the aperture area and the length of the collecting volume, and is independent of *d*. As noted, this result holds for quantities proportional to the tracklength or fluence, such as kerma and exposure, and hence extends the result from our total-tracklength detector to the case of the FAC. Note also that the same result for the effective volume is obtained if the detector volume is offset from the planar aperture (at point P in Fig. 1) by a distance *z*_g_. In that case, *R*_2_ = *R*·[1 + (*z*_g_ + *L*)/*d*], and all appearances of *L*/*d* from Eqs. (11) to (17) are simply replaced by (*z*_g_ + *L*)/*d* which cancel as before.

Taylor [16] obtained the result given in Eq. (17) for a point source using simple geometrical arguments, but required that the fluence be constant over the planar aperture area. The same result was obtained by Aitken [17] for the point source, but because he did not consider Eq. (15) to be a correction factor he interpreted Eq. (17) to hold only for (*R*/*d*)^2^ ≪ 1. In fact, the argument developed above can be further extended to an arbitrary angular distribution of fluence without any restriction on *R*/*d*, with the same result for the effective volume *V*_eff_, but with some other result for the (planar-aperture-average)-to-point correction factor, different from that of Eq. (15).

In the limit of *d* → ∞, the results for a parallel beam are obtained: both correction factors given by Eqs. (15) and (16) are unity, and the effective volume is *V*_eff_ = *V* = π*R*^2^*L*, in complete conformance with the intuitive result. This leads to the interpretation that in a FAC measurement one in effect is simply replacing the divergent beam with an equivalent parallel beam, one with the same fluence rate for the planar aperture. This is an important attribute of a FAC because the effective volume, and hence mass, of air can then be determined quite easily.

The results of a FAC measurement of low-energy photons are then analyzed according to

$$S_{k}=(W/e)\frac{I_{\text{net}}d^{2}}{\rho_{\text{air}}V_{\text{eff}}(1-g)}\prod_{i}k_{i},$$ (18)

where *I*_net_ is the measured net ion current, *d* is the source-to-aperture distance, *ρ*_air_ is the density of air, *V*_eff_ is the product of the aperture area and the length of the collecting volume, the radiative-loss correction *g* is effectively zero for these radiations, and *k_i_* are correction factors for air attenuation, scatter, electron loss, etc.

## 4. The Earlier NBS (Loftus) Exposure Standard for ^125^I Seeds

Loftus [11] performed measurements for three types of ^125^I seeds, all encapsulated in titanium. The encapsulation is in the form of a titanium tube with an outside diameter of 0.8 mm and a wall thickness of 0.05 mm. Welded Ti end-caps seal the seed in the form of a cylinder with rounded ends, with a total length of 4.5 mm. One type of seed incorporated a gold-marker sphere separating two resin spheres on which the radionuclide is adsorbed. A second type is one in which the gold marker sphere is replaced by a ^125^I-coated resin sphere,^2^ and a third type incorporates a silver rod, 3 mm in length, on which is adsorbed ^125^I. These three models comprised all of the ^125^I brachytherapy seeds produced at the time of the measurements. The third type (model 6711) is still manufactured in the United States by Amersham Health.^3^

**Fig. 2:**
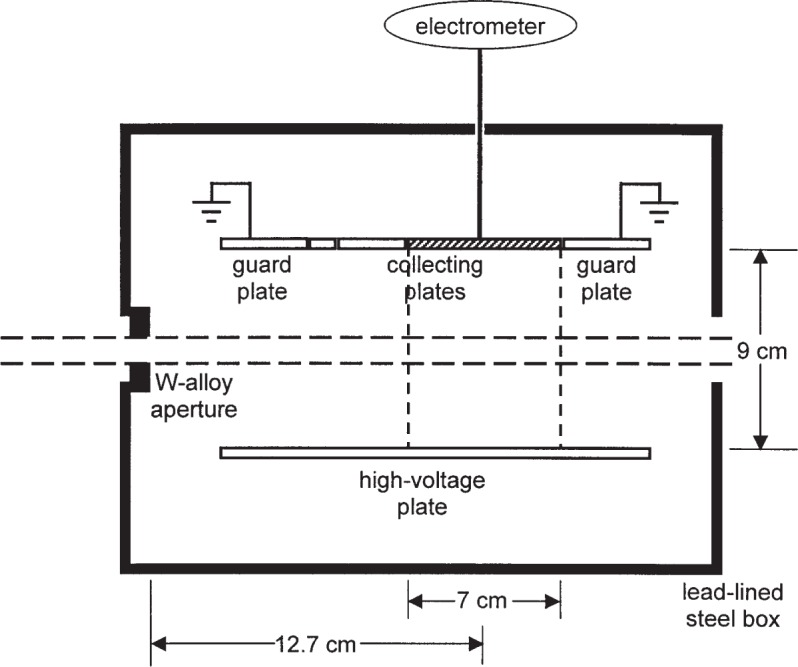
Schematic diagram of the Ritz parallel-plate free-air chamber, viewed from above. The beam enters the chamber from the left. The collecting volume is indicated by the vertical dashed lines at the edges of the 7 cm wide collecting plate (the collecting plates of 1 cm and 3 cm widths, to the left of the cross-hatched plate, are shown also).

Loftus used the standard Ritz [3] free-air chamber (FAC) as the most suitable for the measurement of the radiation from ^125^I. The Ritz parallel-plate FAC is shown schematically in Fig. 2. The aperture diameter is 1 cm; the collector plate^4^ is 7 cm × 9 cm, separated from the high-voltage plate by 9 cm, creating a collecting volume of 567 cm^3^; the air path from the aperture plane to the plane bisecting the 7 cm collector is 12.7 cm. The effective or defined air volume is approximately 5.5 cm^3^, and the mean background current is about 1.6 fA, due primarily to cosmic-ray interactions in the collecting volume. Taking into account the signal strength expected from a single seed, Loftus ensured a sufficiently large signal/background ratio mainly by using arrays of from 4 to 6 seeds per measurement, and using a seed-to-FAC distance of 25 cm. Measurements made also at 50 cm allowed the experimental determination of an apparent attenuation coefficient for the ^125^I radiation in air. Loftus noted that his measured linear attenuation coefficient for air at laboratory conditions was 0.0015 cm^−1^, whereas the coefficient calculated using ^125^I emission spectra and theoretical attenuation coefficients [18] was only 0.0004 cm^−1^. He used the experimental coefficient in his attenuation corrections for the air path from the source to the FAC aperture plane and from the aperture plane through the collecting volume of the FAC.

The measured mean exposure rates for the seed arrays were converted to exposure rates for individual seeds through the transfer of the results to a spherical aluminum re-entrant chamber [9] of outside diameter 20.3 cm. For these sources, the original brass tube insert for the re-entrant chamber was replaced by an aluminum tube with walls 0.64 mm thick and an inside diameter slightly larger than the length of a seed. Thus a seed dropped into the tube will settle horizontally on the bottom of the tube at a position near the center. Multiple drops/measurements were done with the re-entrant chamber to effectively randomize the seed orientation to average over any anisotropy of seed emissions or chamber response. With the long-term stability of the re-entrant chamber checked by a long-half-life ^226^Ra source (see [19]), the calibrated re-entrant chamber would serve as the secondary standard for subsequent routine measurements. The stated uncertainties, expanded with a coverage factor of 2 to approximate that expected at the 95 % confidence level, for the transferred measurements are 3 % for the 6702 seed and 4 % for the 6711 seed [11]. For subsequent re-entrant seed calibrations, the uncertainty in the measurement of the unknown is added, with the typical result for the expanded (95 % confidence level) uncertainty of 5 % for the 6702 seed and 6 % for the 6711 seed [19].

**Fig. 3:**
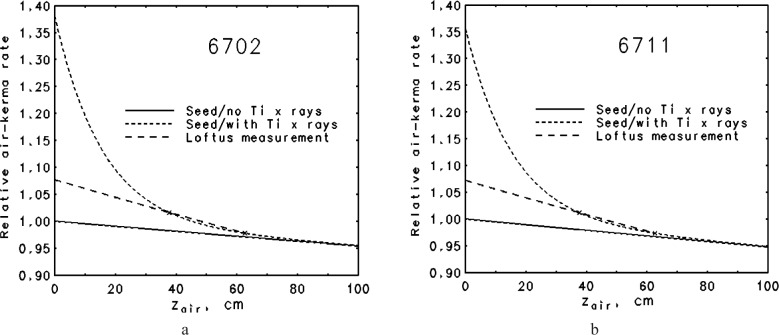
Illustration of the attenuation of the air kerma produced by an ^125^I brachytherapy seed. The solid curve is that due to the emergent ^125^I emissions only; the short-dash curve is that including also secondary Ti characteristic K-shell x rays produced in the Ti encapsulation. The points marked by an × indicate results at two effective distances used by Loftus (1984) to estimate a measured linear attenuation coefficient of 0.0015 cm^−1^, through which the long-dash line has been drawn. These calculated results assume a contribution of Ti x rays that produces the measured attenuation coefficient stated by Loftus (a) 6702 seed, assuming an emergent spectrum with 0.79 % Ti x rays (b) 6711 seed, assuming an emergent spectrum with 0.82 % Ti x rays.

This calibration standard became available in 1985 and has been referred to [20] as the NBS 1985 air-kerma-strength standard, *S_k_*_,1985,std_, for models 6702 and 6711 ^125^I seeds. Soon after the introduction of the standard, Kubo [21] called attention to the influence of the 4.5 keV Ti K x rays on exposure measurements made in air. These Ti x rays are clinically unimportant as they are effectively absorbed by about 1 mm of water, but they could affect the air-kerma strength FAC measurements as done at the NBS. Monte Carlo calculations by Williamson [22] further elaborated on the effects of the Ti x rays on Loftus’ FAC measurements. The situation is illustrated in Fig. 3 in which relative exposure from a parallel beam is plotted as a function of total air path, both for an emission spectrum that includes only the higher energy photons and for one to which an admixture of Ti K x rays has been added. The results are nearly the same for the 6702 seed (Fig. 3a) and for the 6711 seed that also emits secondary Ag K x rays (Fig. 3b). In both cases, the relative probability of Ti x-ray emission (≈0.008) was estimated such that the measured apparent linear attenuation coefficient would be close to the Loftus measured value of 0.0015 cm^−1^. These simplified examples suggest that, by disregarding the contribution by Ti x rays, Loftus significantly overestimated the air-kerma rate compared to that with the Ti x-ray component eliminated.

## 5. The Wide-Angle Free-Air Chamber (WAFAC)

### 5.1 Design

The Ti x rays can be eliminated by a relatively thin Al filter placed between the source and the FAC aperture. However, the need to develop a new instrument to directly measure the air-kerma rate from individual seeds was recognized. One of us (R.L.) designed a new chamber with greatly improved characteristics: (1) The aperture has a diameter of up to 8 cm, and is placed at a distance of nominally 30 cm from the source. This allows the measurement of radiation in a cone with a half-angle of up to approximately 8°, rather than the ≈1° for the Ritz FAC measurements, for an advantage by a factor of more than 40 in solid angle; hence the wide-angle description. (2) The effective or defined volume is ≈704 cm^3^, and the collecting volume is ≈2474 cm^3^, rather than ≈5.5 cm^3^ and 567 cm^3^, respectively, for the Ritz FAC. The larger effective volume makes the WAFAC about 100 times more sensitive than the Ritz FAC. Moreover, the ratio of effective to collecting volumes is about 0.28 for the WAFAC compared to only about 0.01 for the Ritz FAC, giving a much improved signal-to-background ratio.

**Fig. 4:**
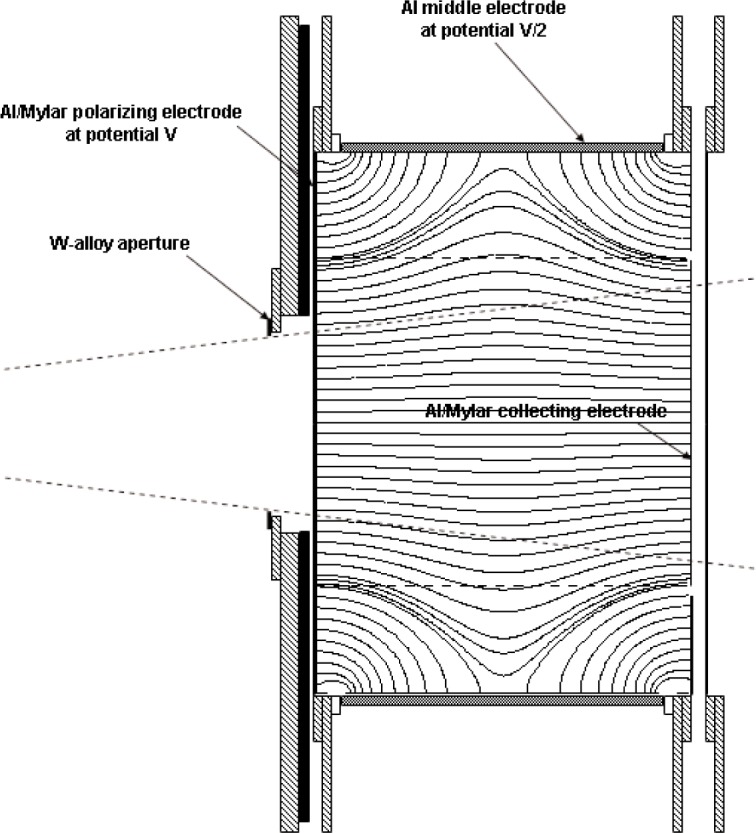
Schematic diagram of original WAFAC with the long middle electrode, showing the electric field lines. Structures of lead are indicated by black, aluminum by gray, and brass by cross hatching.

The design was introduced in 1993 [23]. The WAFAC is a cylindrical chamber with circular symmetry about the beam axis. The WAFAC itself consists basically of: (a) a front, circular-area, aluminized-Mylar^5^, high-voltage electrode, held at a potential *V*; (b) a back, circular-area, aluminized-Mylar electrode on which the aluminum has been etched away along a narrow-width circle, dividing the foil into a central circular collecting electrode and an annular guard ring, both at ground potential; (c) a cylindrical aluminum middle electrode separating the front and back electrodes, held at potential *V*/2 to shape the electric field; and (d) mechanical support and auxiliary measurement instrumentation (electrometer, air temperature and pressure probes, etc.). The addition of a source-positioning device^6^ and of aluminum foils to absorb the Ti x rays completes the measurement system. Figure 4 shows a schematic diagram of the original WAFAC, indicating the major components and the measurement geometry for which it was designed. The radius of the collecting electrode is larger than that of the intersecting conical-beam trace by an amount (≈1.1 cm) to ensure that effectively all the ionization from secondary electrons produced by unscattered photons is collected.

**Fig. 5:**
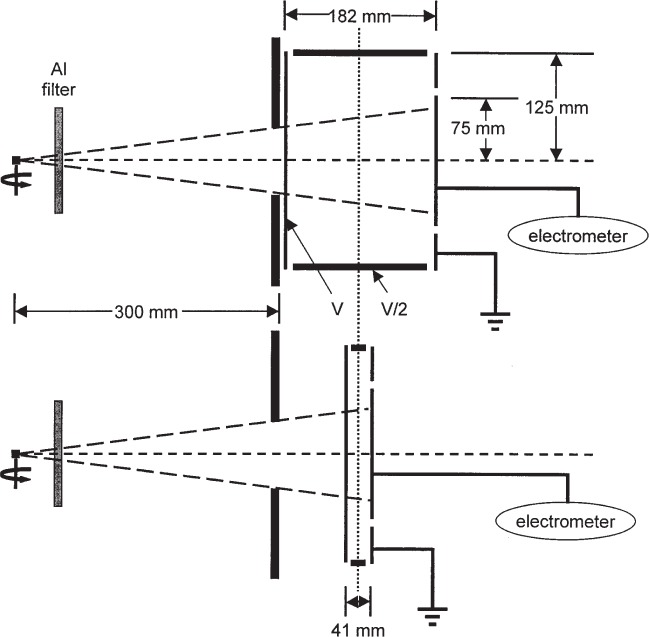
The WAFAC measurement scheme, involving the subtraction of the results of a second measurement using the small chamber length in order to remove any possible effects due to the presence of the front and back aluminized-Mylar electrodes. The middle electrode lengths shown are for the original WAFAC.

The front and back electrodes of aluminized Mylar, about 1 mg/cm^2^ thick, intersect the beam. Secondary electrons produced by photon interactions in the aluminized-Mylar films are not characteristic of those created in air, but—due to their short range—are confined to regions near the Mylar films. Any potential perturbing effects of the aluminized-Mylar electrodes are removed by subtracting the charge measured for a small chamber length from that for a large chamber length, keeping constant the air path from the aperture plane to the center plane of the collecting volume. This design, illustrated in Fig. 5, ensures that the WAFAC measurements are equivalent to those of a free-air chamber whose effective volume is the aperture area times the difference in the lengths of the collecting volumes. Figure 5 shows the presence of the Al filter, and also indicates that the seed is rotated about its long axis during the measurement to effectively average over any axial non-uniformity in air-kerma rate^7^. Four middle electrodes were constructed for different collecting-volume lengths, as given in Table 1. The lengths of the actual collecting volumes are very close to 3.0 cm larger due to the dimensions of electrode fixtures and insulating gaps. However, the effective volume is determined only by the difference in middle electrode lengths, typically those of the largest (15 cm) and smallest (1 cm). Regardless of which middle electrode is used, the length of the air path from the aperture plane to the center plane of the collecting volume is kept the same as that for the case of the longest middle electrode: one-half the collecting-volume length of 18.25 cm plus a gap of 1.53 cm from the aperture plane to the front electrode in the geometry shown in Fig. 5.

The electric-field lines in the WAFAC for the 18.25 cm collecting length are shown in Fig. 4. Potentials on a fine grid (every 0.5 mm) were obtained from an adaptive-mesh finite-elements calculation for the appropriate symmetric geometry using Ansoft’s Maxwell 2D Field Solver for the electrostatic problem. The electric-field vectors were then calculated from the potentials, using cubic-splines to interpolate and obtain the needed partial derivatives. The results indicate that virtually all ionization within the ≈1.1 cm margin of the conical beam is collected. There is noticeable bulging of the field lines toward the cylindrical middle electrode, but this is of importance only in the determination of the actual collecting volume for the purpose of correcting for the small contribution of ionization from photons scattered in the chamber. Similar calculations were done for the 4.12 cm length, the results of which indicate essentially straight electric field lines for all radii of interest.

W-alloy^8^ apertures, 1 mm thick, were constructed with diameters ranging from 1 cm to 8 cm. Tests showed that the air-kerma strength measured with the WAFAC was independent of aperture size. The results of the WAFAC were compared to those of the Ritz FAC for four different NIST low-energy x-ray beam qualities (see Table 2); both chambers were used with 1 cm diameter apertures. The level of agreement is shown in Table 2; the mean of the ratios of the 12 measurements is 1.003 ± 0.003 (one standard deviation), demonstrating very good agreement.

For our routine measurements, a new 8 cm diameter W-alloy aperture was made that is 4 mm thick to ensure negligible penetration by the gamma rays emitted in ^125^I and ^103^Pd decay. Seed measurements are based on the use of the 15 cm and 1 cm middle electrodes (18.25 cm and 4.12 cm collecting lengths) to maximize the net collecting volume and hence the signal. For the 18.25 cm collecting length, the high-voltage electrode is typically held at 2000 V, and at 450 V for the 4.12 cm length, to maintain similar field strengths of about 110 V/cm. Table 3 gives the magnitude of the leakage and background currents for the WAFAC. The leakage current is surface leakage, practically identical for the two electrodes and independent of applied voltage; it varies slowly with time, probably due to changes in humidity, but is seldom larger than 100 fA. The background currents, whose absolute values are independent of polarity, are reasonably consistent with the expected ionization rate due to cosmic rays at sea level, which would produce ≈1 aA/cm^3^.

**Fig. 6:**
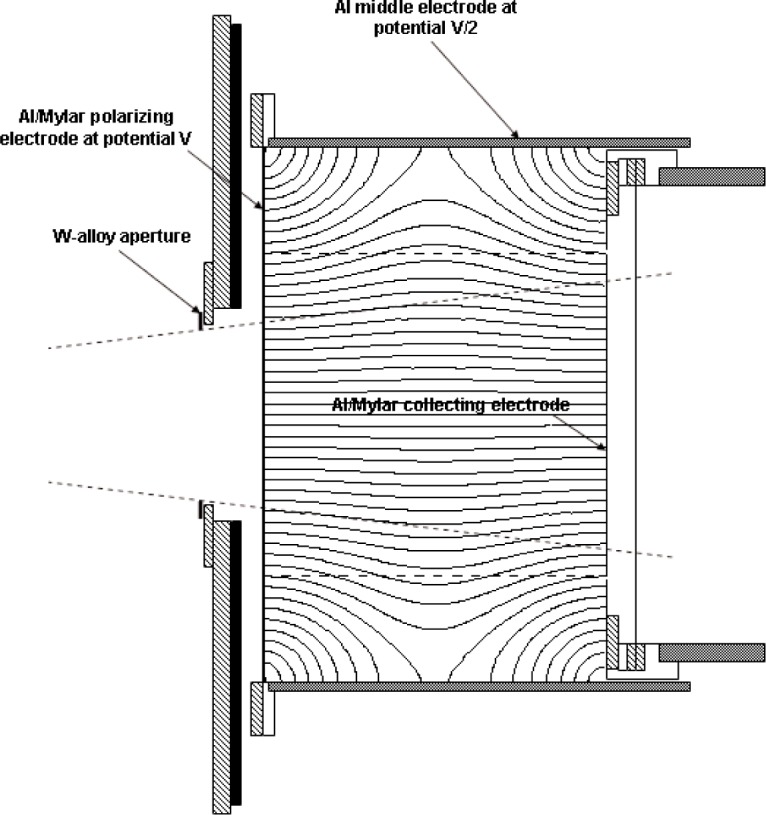
Schematic of automated WAFAC extended to the long middle electrode, showing the electric field lines. Structures of lead are indicated by black, aluminum by gray, and brass by cross hatching.

An automated version of the WAFAC was constructed that allows for computer-controlled, motor-driven, variable middle-electrode lengths, while holding fixed the positions of both the aperture plane and the center plane of the collecting volume. A schematic of the chamber is given in Fig. 6. This chamber is used to measure the net charge for the difference in collecting lengths of 16.0 cm and 4.3 cm, for which the high-voltage electrode is held at 1670 V and 450 V, respectively. The electric field lines for this chamber with the expanded 16 cm collecting length are also shown in Fig. 6, and again indicate that virtually all the charge produced by primary photon interactions is collected. The relevant field lines are nearly straight for the contracted 4.3 cm collecting length used to remove the perturbing effects of the front and back electrodes. The air path from the aperture plane to the center plane of the collecting volumes is, in this case, held at one-half the largest collecting volume length of 16.0 cm plus a gap of 2.12 cm from the aperture plane to the front electrode. Results from both WAFACs have been compared for a large number of seeds of various designs, showing agreement to within 0.5 %.

**Fig. 7:**
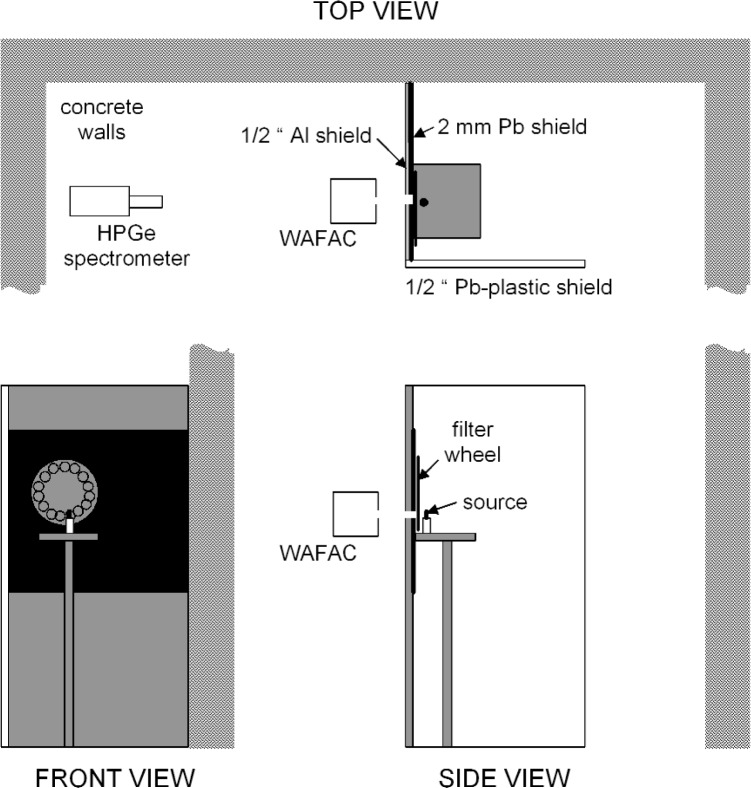
Layout of brachytherapy seed measurement structures. The schematic is approximately to scale. The front view is from inside the seed enclosure, looking out toward the WAFAC. The side view is from outside the seed enclosure.

A walk-in enclosure was constructed to house the source-positioning fixture. The layout is shown in Fig. 7. In addition to providing for personnel safety during measurements, the enclosure effectively pre-collimates the beam to be measured, thereby reducing significantly the photons scattered in air about the source that can enter the FAC. The source is held^9^ with its axis vertical on the end of a vertical thin (1.5 mm OD) nylon rod (length about 1.3 cm) fixed on a conical nylon base (1.3 cm high, with a base of ≈1.3 cm OD), attached to a motor-driven (≈1 rpm) cylindrical shaft of Micarta^10^ (3.5 cm OD and 16.5 cm high) that is fastened to an aluminum tray (44.5 cm × 44.5 cm × 0.6 cm) whose height is adjustable. The seed is thus held at about 1.56 m above a concrete floor, and is nearly enclosed by barriers. In the direction of the WAFAC there is a 1.3 cm thick, 1.22 m wide, and 2.44 m tall aluminum barrier, lined on the seed side from about 1.0 m to 2.1 m from the floor with 2 mm of Pb. A Pb-lined circular portal, with a net diameter of 4.7 cm and whose center is about 1.56 m above the floor, allows a direct beam to pass through to the WAFAC. Concrete room walls (≈4.6 m tall) form two more barrier sides, and a 1.22 m × 2.44 m tall leaded-plastic plexiglass shield (1.2 cm thick and 0.2 mm Pb equivalent) forms the fourth side, leaving an opening about 85 cm wide for access. A motor-driven filter/shutter wheel is mounted on the seed side of the Pb/Al barrier. The Al wheel is ≈1.3 cm thick and 48 cm in diameter with 15 holes (each of 6.4 cm diameter) equally spaced and near the edge in which are mounted a 1.3 cm thick Pb shutter and a series of Al filters. The seed position is about 8.4 cm from the Pb surface of the Pb/Al barrier and about 6.9 cm from the plane of the Al filters. One of the holes in the filter/shutter wheel accommodates a ≈150 MBq ^241^Am source used for periodic constancy checks of the response of the two WAFACs.

### 5.2 Correction Factors

The determination of the air-kerma strength from the measurements proceeds according to Eq. (18), slightly reinterpreted for the WAFAC as

$$S_{k}=(W/e)\frac{I_{\text{net},\text{dif}}d^{2}}{\rho_{\text{air}}V_{\text{eff},\text{diff}}(1-g)}\prod_{i}k_{i},$$ (19)

where here *I*_net,dif_ is the difference in net current (signal minus background and leakage) for the large and small collecting volumes, and *V*_eff,dif_ is the aperture area times the difference in the lengths of the large and small collecting volumes.

The evaluation of a number of correction factors is relatively straightforward. Using temperatures and pressures measured with calibrated instruments, the density of dry air during the measurement is corrected according to

$$\rho_{\text{air}}=\rho_{0}\frac{273.15{}^{\circ}C+T_{0}}{273.15{}^{\circ}C+T_{\text{air}}}\frac{P_{\text{air}}}{P_{0}},$$ (20)

where *ρ*_0_ = 1.196 mg/cm^3^ for *T*_0_ = 22 °C and *P*_0_ = 1.01325 kPa. However, results are reported for reference conditions of *T*_0_ = 22 °C and *P*_0_ = 1.01325 kPa. Further corrections for the effects of humidity on the density of air are considered later.

The correction to a reference date and time is done on the basis of the assumed decay of the stated radionuclide, as no assessment of impurities is performed. Conventional half-lives have been used: (a) 59.43 d for ^125^I, and (b) 16.991 d for ^103^Pd.

Corrections for the recombination of ions and electrons before they are collected in the WAFAC were determined using multiple-voltage-extrapolation methods described in Lamperti et al. [24] and based on the work of Scott and Greening [25]. The correction factor is evaluated as *k*_sat_ = 1.0 + *a*_sat_*I*, where *a*_sat_ = 3.12 × 10^8^ A^−1^ applies to both WAFACs, and *I* is the current measured in either the large or small collecting volumes before subtracting background and leakage currents.

The correction factor for converting the results for the planar-aperture area to the point value is given for the point-isotropic source by Eq. (15). For our aperture radius *R* of 4.0 cm and our measurement distance *d* of 30.0 cm, the correction factor is *k*_invsq_ = 1.0089. There is evidence that due to their internal structure the brachytherapy seeds do not approximate a point-isotropic source at our measurement distance, with some designs perhaps differing considerably (see, e.g., [26]). Although our calibration reports clearly indicate that the measurements represent the average over the conical beam defined by our aperture radius and measurement distance (i.e., a half-angle of ≈7.6°), the correction *k*_invsq_ for the point-isotropic source is applied in all cases. One can either (a) accept the result as a useful calibration quantity, (b) remove the point-isotropic value of *k*_invsq_ from the result to render it a true average quantity, or (c) replace the value of *k*_invsq_ with one applicable to the particular source design to obtain a more accurate point result.

A number of correction factors are in principle dependent on the emergent photon spectrum, but in practice appear to be nearly the same for both ^125^I and ^103^Pd photons. The so-called electron-loss correction, to account for that portion not counted of ionization from secondary electrons produced by primary photons in the FAC, is by design of the chamber *k*_elec_ = 1.0.

A small contribution to the measured air kerma is from photons scattered by the nylon post on which the sources are mounted. Contributions from scatter by air and other materials present in the measurement are considered separately. The post-scatter correction was determined by measurements with and without a second, dummy post held on the seed’s top end. The value *k*_post_ = 0.9985 was found, with differences among seed designs and spectra falling within measurement uncertainties.

The defining plane of the right-circular aperture that admits the photons is assumed to be the plane farthest from the source. The full thickness of the aperture plate and associated fixtures is large enough^11^ to mostly stop the incident photons, so that the penetration of the primary photons^12^ through the inner cylindrical surface of the aperture is of primary concern. Based on air-kerma attenuation calculations that consider the point-isotropic source emitting the appropriate spectra of photons, it has been found that for our measurement geometry *k*_pen_ = 0.9999 can be applied to the ^125^I and, as well, to the ^103^Pd seeds for photons with energies ≲40 keV. The small contribution of the 357.4 keV and 497.1 keV gamma rays emitted in ^103^Pd decay that penetrate the plates slightly reduce the correction factor for that radionuclide to *k*_pen_ = 0.9997.

Other correction factors are more sensitive to the spectrum of photons emerging from the source. Because we have not been completely successful in determining most of these factors through measurements, theoretical estimates for them have been obtained and confirmed experimentally when possible. The tracklength approach introduced in Section 3 can be generalized into a description of the measurement of a point-isotropic source. Then the expected ionization current in the WAFAC can be evaluated approximately as

$$\begin{array}{c} I\approx \frac{1}{W/e}\sum_{j}\frac{{\dot{N}}_{j}}{4\pi }\int_{0}^{2\pi }d\omega \int_{\cos\theta_{c}}^{1}d(\cos\theta )\exp\{-[\mu_{\text{air}}(E_{j})z_{\text{air}}\\ +\mu_{A1}(E_{j})z_{A1}+\mu_{\text{air}}(E_{j})z_{g}]/\cos\theta \}\\ \times \int_{0}^{L}\frac{dz}{\cos\theta }\exp\{-\mu_{\text{air}}(E_{j})z/\cos\theta \}E_{j}\mu_{\text{en}}(E_{j}), \end{array}$$ (21)

where ${\dot{N}}_{j}$ is the emission rate of the photon with energy *E_j_*, *µ* is the linear attenuation coefficient for the indicated material, *z*_Al_ is the Al absorber-foil thickness, *z*_air_ + *z*_Al_ = *d* (i.e., the distance from source axis to the aperture plane), *z*_g_ is the air-gap thickness (from the aperture plane to the front plane of the collection volume), *L* is the length of the cylindrical collection volume, *µ*_en_ is the energy-absorption coefficient for air, and cos*θ*_c_ = [1 + (*R*/*d*)^2^]^−1/2^ with *R* the aperture radius as before. Contributions to the ionization current from either photon scatter within the collection volume or photon scatter outside the collection volume have been ignored in Eq. (21), but will be considered separately. The result of the integrations is

$$I\approx \frac{1/2}{W/e}\sum_{j}{\dot{N}}_{j}E_{j}\mu_{\text{en}}(E_{j})J_{j},$$ (22)

where for the general result (*µ*_air_*L* ≠ 0)

$$J_{j}=\frac{1}{\mu_{\text{air}}(E_{j})}(M_{1}-M_{2}),$$ (23)

with

$$M_{k}=b_{k}\left\{\frac{\exp(-b_{k})}{b_{k}}-E_{1}(b_{k})-\left[\frac{\exp(-b_{k}/\cos\theta_{c})}{b_{k}/\cos\theta_{c}}-E_{1}(b_{k}/\cos\theta_{c})\right]\right\},$$ (24)

*b*_1_ = *µ*_air_(*E_j_*)*z*_air_ + *µ*_Al_(*E_j_*)*z*_Al_ + *µ*_air_(*E_j_*)*z*_g_, *b*_2_ = *b*_1_ + *µ*_air_(*E_j_*)L, and $E_{1}(x)=\int_{x}^{\infty }dt\frac{e^{-t}}{t}$ is the exponential integral.

With *I* being the general result, the needed corrections can be estimated by successively setting factors equal to zero. For example, for the effects of attenuation of the primary beam in the air path from the aperture plane through the WAFAC volume, the last terms in both *b*_1_ and *b*_2_ are set to zero so that ${b^{\prime }}_{1}=\mu_{\text{air}}(E_{j})z_{\text{air}}+\mu_{A1}(E_{j})z_{A1}$ and ${b^{\prime }}_{2}={b^{\prime }}_{1}$. For this case, Eqs. (23) and (24) cannot be used, but integration of Eq. (21) gives instead

$${J^{\prime }}_{j}=L[E_{1}({b^{\prime }}_{1})-E_{1}({b^{\prime }}_{1}/\cos\theta_{c})],$$ (25)

which when used in Eq. (22) gives the expected current without air attenuation beyond the aperture plane. The corresponding correction factor is then evaluated as *k*_att-WAFAC_ = *I*′/*I*. For the NIST measurements, *k*_att-WAFAC_ is estimated to vary from about 1.004 to 1.009, depending on the emergent photon spectrum.

Continuing, for the effects of attenuation of the primary beam in the air path from the source to the aperture plane, one sets ${b^{\prime \prime }}_{1}=\mu_{A1}(E_{j})z_{A1}$ and ${b^{\prime \prime }}_{2}={b^{\prime \prime }}_{1}$, and obtains the result of Eq. (25) with double primes instead of single primes. The correction factor is evaluated as *k*_att-SA_ = *I*′′/*I*′. For the NIST measurements, *k*_att-SA_ is estimated to vary from about 1.012 to 1.027, depending on the emergent photon spectrum.

Finally, for the effects of the attenuation of the primary beam by the Al absorber foil, ${b^{\prime \prime \prime }}_{1}={b^{\prime \prime \prime }}_{2}=0$, $J^{\prime \prime \prime }=L\ln\left(\frac{1}{\cos\theta_{c}}\right)$, and the correction *k*_foil_ = *I*‴/*I*″. The Al foil used to absorb the Ti K x rays in the NIST routine measurements has a thickness of 0.008636 cm. The *k*_foil_ correction is the largest one involved in our measurements, varying from about 1.03 for the harder emergent spectrum from ^125^I seeds to about 1.08 for the softer emergent spectrum from ^103^Pd seeds.

To evaluate correction factors using Eqs. (22)–(25), photon narrow-beam total attenuation coefficients (i.e., including coherent scattering) were taken from Berger and Hubbell [27], and photon mass energy-absorption coefficients were taken from Seltzer [28] and Seltzer and Hubbell [29]. Spectra of the line emissions from a variety of seeds have been obtained by deconvolving pulse-height distributions measured with a high-purity Ge spectrometer [30] using knowledge of the detector response function. The results have been averaged to obtain representative relative emission rates for six categories of seeds, depending on the incorporated radionuclide and the relative strength of significant secondary characteristic x-ray emission induced in structural materials (Ag and Pd). The representative spectra are given in Table 4. The spectra are only nominal; there have been noticeable variations among seeds in the same category, even from the same manufacturer. The use of as many as 4 significant figures in Table 4 is only to insure that the relative spectra explicitly sum to unity. These spectra of photons emerging from the encapsulated seeds can be compared with the basic emission data for the radionuclide decay given in Table 5. Note that although the average energy for ^103^Pd turns out to be the same for the photons emergent from the extended source as from the basic emission data, the relative spectra are not.

**Fig. 8:**
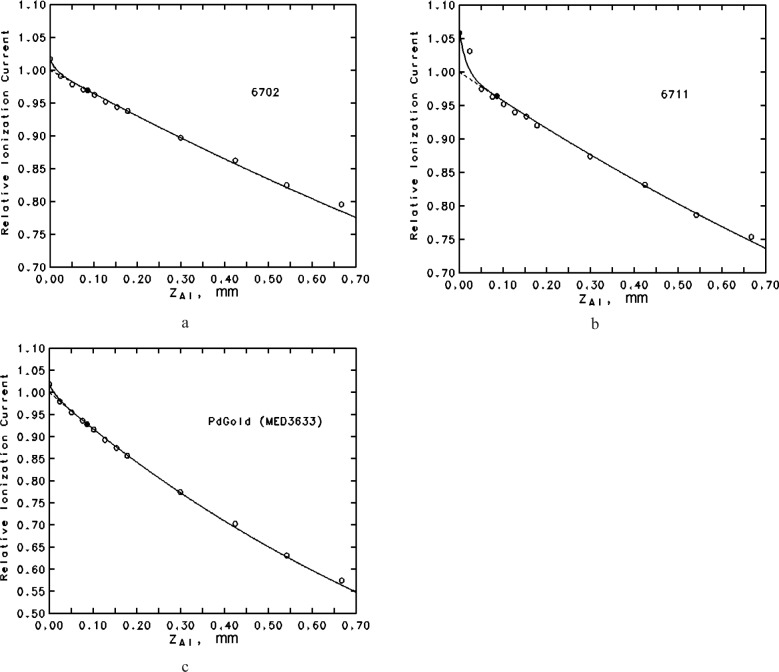
Transmission curves in terms of ionization current measured in the WAFAC as a function of Al foil absorber thickness. The points are values measured using the long collection length; the filled point indicates the standard absorber. The dashed curve is from calculations according to the text, based on the appropriate emergent spectrum given in Table 4; the solid curve is from calculations including a contribution of Ti K-shell characteristic x rays produced in the encapsulation (a) 6702 ^125^I seed, assuming an emergent spectrum with 0.42 % Ti x rays (b) 6711 ^125^I seed (emits also secondary Ag x rays), assuming an emergent spectrum with 1.81 % Ti x rays (c) PdGold (MED3633) ^103^Pd seed, assuming an emergent spectrum with 0.85 % Ti x rays.

The ability of Eq. (21) to predict measured relative ionization currents as a function of Al absorber thickness is illustrated in Fig. 8 for three seeds: one with only ^125^I emissions, one with ^125^I and secondary Ag K x rays, and one with only ^103^Pd emissions. In addition to the assumed relative emission probabilities for photons of energies greater than 10 keV, an admixture of the ≈4.5 keV Ti lines was included to make the transmission curve more realistic for very thin absorbers. The agreement between the measured and calculated results is deemed sufficient to confirm the accuracy of the calculated foil-attenuation correction factor, and so to extend the theoretical estimates to the smaller air-attenuation correction factors for which measurements have not produced any useful results.

**Fig. 9:**
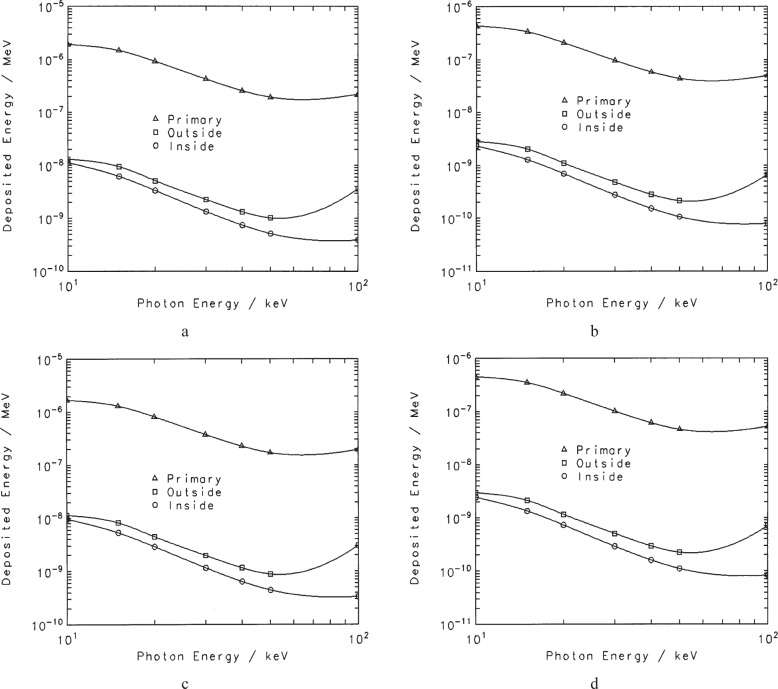
Contributions to the ionization current from the primary photon (triangles), from photons scattered outside the WAFAC (squares), and from photons scattered inside the WAFAC (circles). Results are from Monte Carlo calculations for monoenergetic sources in the WAFAC measurement geometry (a) Original WAFAC with the long collection length (b) Original WAFAC with the short collection length (c) Automated WAFAC with the long collection length (d) Automated WAFAC with the short collection length.

The attenuation of the mostly monoenergetic^13^ photons emerging from the source is taken into account by the correction factors defined above. Although attenuation at the energies of interest is mainly through photoelectric absorption, there is some scatter of the photons in the air (and other material) between the source and the aperture (i.e., outside the chamber). The contribution to the measured ionization current of this component must be subtracted to produce the result for a source in vacuum. Additionally, the contribution to the measured ionization current from photons scattered within the chamber must be removed in the realization of air kerma or exposure. Both of these contributions were estimated from the results of a series of Monte Carlo calculations that simulated the measurement in realistic detail. Using the CYLTRAN code in the Integrated Tiger Series [33], the WAFACs were both modeled in their long-collecting-length configurations, with the 8 cm diameter aperture structure. The calculations were done for a point-isotropic source (30 cm source-to-aperture-plane) of monoenergetic photons with energies from 10 keV to 500 keV. One series of calculations was done for the source suspended in vacuum, but with air included from the aperture plane through the WAFAC. A second series included also the main features of the seed enclosure, the Al absorber foil, the external air, and the surrounding concrete room structures. Results of the first series give information on the internal-scatter effects, and the second on both the internal and the external scatter, allowing the separation of the two components. The photon fluence was scored in regions within the WAFAC volume and converted to absorbed dose in the air through the use of photon mass energy-absorption coefficients. These calculations facilitated the development of results for the various collecting lengths, including the bulges^14^ in the collecting volume indicated by the field lines in Figs. 4 and 6. Results for the energy deposited within the relevant collecting volumes are shown in Fig. 9 for the components of interest. Interpolating the results in Fig. 9 for the appropriate emission spectra, the correction factors for the effects of internally scattered photons are calculated according to

$${k^{\prime }}_{\text{int-scatt}}(V)=\frac{1}{1+\sum_{j}{\dot{N}}_{j}\varepsilon_{\text{int-scatt}}(E_{j},V)/\sum_{j}{\dot{N}}_{j}\varepsilon_{\text{primary}}(E_{j},V)},$$ (26)

where *ε*_int-scatt_(*E_j_*,*V*) is the energy deposited in the collecting volume *V* by internally scattered photons for primary photons of energy *E_j_*, and *ε*_primary_(*E_j_*,*V*) is the energy deposited by the primary photons themselves. Similarly

$${k^{\prime }}_{\text{ext-scatt}}(V)=\frac{1}{1+\sum_{j}{\dot{N}}_{j}\varepsilon_{\text{ext-scatt}}(E_{j},V)/\sum_{j}{\dot{N}}_{j}(\varepsilon_{\text{int-scatt}}(E_{j},V)+\varepsilon_{\text{primary}}(E_{j},V))},$$ (27)

where *ε*_ext-scatt_(*E_j_*,*V*) is the energy deposited in the collecting volume *V* by externally scattered photons for primary photons of energy *E_j_*. The values of *k*′ obtained for the large (long) and small (short) collecting volumes differ only in the fourth significant figure for both the internal- and external-scatter corrections, so for convenience an effective value *k* = [*k* ′(*V_L_*)*V_L_* − *k*′(*V_s_*)*V_s_*]/(*V_L_* − *V_s_*), where *V_L_* and *V_S_* are the large and small volumes, is applied to the net ionization current. The effective internal-scatter correction factor *k*_int-scatt_ is about 0.996 to 0.997 depending on the photon spectrum, and the effective external-scatter correction factor *k*_ext-scatt_ is about 0.994 to 0.995 depending on the photon spectrum. Note that the effects of external scatter are much smaller than predicted by the usual build-up factors for air because of the partial shielding between the source and the WAFAC.

Humidity affects the results of the free-air chamber measurements in a number of ways. In principle, the photon attenuation coefficients for moist air are different from those for dry air. However, over the range of conditions pertinent to NIST measurements, the effect on the various air-attenuation correction factors appears to be negligible. Depending on the water-vapor content, there can be small changes in the photon mass energy-absorption coefficient for air, the density of the air, and the *W*/*e* value for air. For the combined effects of these small changes, a humidity correction factor has been calculated [34] as

$$k_{\text{humidity}}=\frac{\rho_{\text{dry-air}}}{\rho_{\text{humid-air}}}\frac{W_{\text{humid-air}}}{W_{\text{dry-air}}}\frac{\sum_{j}{\dot{N}}_{j}E_{j}{\left(\mu_{\text{en}}/\rho \right)}_{\text{dry-air}}}{\sum_{j}{\dot{N}}_{j}E_{j}{\left(\mu_{\text{en}}/\rho \right)}_{\text{humid-air}}}.$$ (28)

**Fig. 10:**
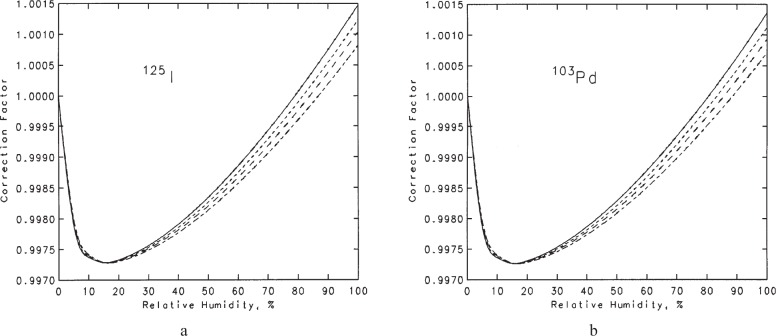
Humidity corrections for free-air chamber measurements of the air kerma from low-energy brachytherapy sources. Solid curve: *T* = 23 °C, *P* = 745 mm Hg. Short-dash curve: *T* = 23 °C, *P* = 770 mm Hg. Long-dash curve: *T* = 22 °C, *P* = 745 mm Hg. Long-short-dash curve: T = 22 °C, *P* = 770 mm Hg. (a) ^125^I seeds (b) ^103^Pd seeds.

The density of humid air was calculated using the equation of Giacomo^15^ [35], which takes into account the small CO_2_ content, the compressibility of the air-water-vapor mixture, and the enhancement factor (that expresses the fact that the effective saturation vapor pressure of water in air is greater than the saturation vapor pressure of pure vapor phase over a plane of pure liquid water). The variation of *W*_humid-air_/*W*_dry-air_ as a function of the partial pressure of water vapor was taken from the curve in Ref. [34] based on the results of Niatel [38]. Generally, the result for *k*_humidity_ is a complex function of temperature, pressure, relative humidity, and photon spectrum. The correction factor as a function of relative humidity, for temperatures of 22 °C and 23 °C and for pressures of 745 mm Hg and 770 mm Hg, are shown in Fig. 10a for the ^125^I spectrum and in Fig. 10b for the ^103^Pd spectrum. The temperatures and pressures chosen for these graphs have been judged to cover the measurement environment encountered in the NIST laboratory. The relative humidity in the laboratory (for which only an imprecise measurement is made) usually can vary from ≈15 % to ≈55 %. Considering the restricted range of values for these limits, it was deemed sufficient to simply use a mean value and to consider deviations as an uncertainty. It turns out that the mean value is 0.9979 for all the seed spectra considered. This value, essentially 0.998, is the same as the humidity correction used for NIST free-air-chamber measurements of air kerma from our x-ray beams.

A summary of the values of correction factors derived from the analysis outlined above is given in Table 6. The accuracy of our determination of the correction factors is judged to be less than implied by the number of significant figures given in Table 6; they are carried to help avoid round-off effects on the product. Table 6 also includes correction factors derived from an earlier, somewhat less-refined analysis. However, it is important to note that these earlier correction factors, developed for the start of our calibrations on 1 January 1999, are still being used in order to maintain consistency with published coefficients to convert air-kerma strength to reference absorbed dose in water used in clinical dosimetry protocols. The differences between the currently implemented values and the more refined values are not significant (< 0.5 %) for most seed types, except perhaps for ^125^I seeds with the largest contributions of Ag or Pd K x rays.

## 5.3 Uncertainties

Because the strength of individual seeds can vary significantly, the Type A uncertainty^16^ for the net current *I*_net,diff_ is calculated as the standard deviation of the mean, *s_I_*, from replicate measurements for each calibration. The contributions to uncertainty in the determination of the air-kerma strength with the WAFAC for the remainder of the components have been estimated and are given in Table 7, to be effective 1 January 2004. Note that, with this approach, the combined total (Type A + Type B) standard uncertainties can be evaluated as ${\left(s_{I}^{2}+0.762^{2}\right)}^{1/2}$ for ^125^I seeds, and as ${\left(s_{I}^{2}+0.728^{2}\right)}^{1/2}$ for ^103^Pd seeds.

## 6. Relationship to the Earlier NBS Standard

Differences between the Loftus [11] standard and the WAFAC standard are pertinent only for the 6702 and 6711 ^125^I seeds that are common to both measurements. The differences, established during the testing of the original WAFAC, are due largely to the effect of the Ti K x rays on the Loftus estimate of the air-attenuation correction. The ratio of the new NIST WAFAC-based to the previous standard were determined by measurements of the same seed both with the WAFAC and with the spherical re-entrant chamber to which the Loftus measurements were transferred. The results, given in Table 8, were communicated to the medical physics community during the introduction of the WAFAC standard [20,39,40].

## Figures and Tables

**Table 1:** Pertinent dimensions of the original WAFAC. Uncertainties are standard deviations of length measurements sampled about the circumference of the cylindrical electrodes

Length of middle electrode (cm)	Length of collecting volume (cm)
15.2513±0.0005	18.25
7.5795±0.0040	10.58
4.1673±0.0055	7.17
1.12103±0.0002	4.12

**Table 2:** Ratio of measured charge per unit effective volume: WAFAC to Ritz FAC. The charge measured with the 1 cm middle electrode was subtracted to remove effects of the front and back electrodes, as discussed in the text. Results are given for four x-ray beam qualities indicated by the NIST beam code

Beam code^a^	Net collecting-volume length (cm)		
	3.05	6.46	14.13
M20	1.004	1.005	1.008
M30	1.002	0.998	0.994
H30	1.004	1.007	1.002
L40	1.004	1.008	1.004

a X-ray beams are from W anodes; in the NIST beam codes, the letter indicates light (L), moderate (M) or heavy (H) filtration, and the number is the constant potential in kilovolts. Further details can be found at http://ts.nist.gov/ts/htdocs/230/233/calibrations/ionizing-rad/x-gamma-ray.htm#46010C.

**Table 3:** Approximate leakage and background currents for the WAFAC

	Length of collecting volume (cm)	
	4.12	18.25
Leakage	10 fA	10 fA
Background	±3 fA	±15 fA

**Table 4:** Relative energy spectra of photons emergent in the transaxial direction from prostate seeds, derived from HPGe spectrometry

		Energy keV	^125^I seed^a^	^125^I+ 0.053 Ag Kx seed^b^	^125^I+ 0.094 Ag Kx seed^c^	^125^I+ 0.195 Ag Kx seed^d^	^125^I+ 0.181 Pd Kx seed^e^	^103^Pd seed^f^
^125^I emissions	γ	35.49	0.0521	0.0493	0.0472	0.0419	0.0426	
	Te Kβ_2,4_	31.70	0.0347	0.0329	0.0315	0.0280	0.0285	
	Te Kβ_1,3,5_	30.98	0.1556	0.1473	0.1410	0.1253	0.1274	
	Te Kα_1_	27.473	0.4981	0.4717	0.4512	0.4009	0.4079	
	Te Kα_2_	27.202	0.2595	0.2458	0.2351	0.2089	0.2126	
Ag K x rays	Kβ_2,4_	25.46		0.0024	0.0043	0.0089		
	Kβ_1,3,5_	24.94		0.0094	0.0166	0.0345		
	Kα_1_	22.163		0.0281	0.0499	0.1034		
	Kα_2_	21.990		0.0131	0.0232	0.0482		
Pd K x rays	Kβ_2,4_	24.30					0.0068	
	Kβ_1,3,5_	23.81					0.0308	
	Kα_1_	21.177					0.1003	
	Kα_2_	21.020					0.0431	
^103^Pd emissions	γ	39.76						0.0016
	Rh Kβ_2,4_	23.17						0.0321
	Rh Kβ_1,3,5_	22.72						0.1731
	Rh Kα_1_	20.216						0.5620
	Rh Kα_2_	20.074						0.2312

Mean Energy (keV)			28.51	28.21	27.97	27.39	27.28	20.74

a Assumed for Nycomed-Amersham 6702, North American Scientific / Mentor IoGold (MED3631-A/M), Bebig / UroMed Symmetra I-125, International Brachytherapy Intersource^125^, SourceTech Medical STM1250, Best Medical International I-125.

b Assumed for Implant Sciences I-Plant.

c Assumed for DraxImage BrachySeed.

d Assumed for Nycomed-Amersham 6711, International Isotopes Inc. / Imagyn IsoSTAR, Mills Biopharmaceuticals / UroCor ProstaSeed, Eurotope I-125, IsoAid I-125.

e Assumed for Syncor PharmaSeed.

f Assumed for Theragenics / Indigo Medical TheraSeed 200, North American Scientific PdGold (MED3633), International Brachytherapy InterSource^103^, Bebig, Best Medical International Pd-103.

**Table 5:** Decay/emission data as compiled from references [30] and [31]. Photon mass total attenuation coefficients *µ*/*ρ*, mass energy-transfer coefficients *µ*_tr_/*ρ*, and mass energy-absorption coefficients *µ*_en_/*ρ* from Seltzer [27] and Seltzer and Hubbell [28]

^125^I Decays by electron capture *T*_1/2_ = 59.40 ± 0.01 d Limiting specific activity = 6.51 × 10^14^ Bq/g (1.76 × 10^4^ Ci/g)						
	Energy (keV)	Photons per disintegration	(*µ*/*ρ*)_air_ (cm^2^/g)	(*µ*_tr_/*ρ*)_air_ (cm^2^/g)	(*µ*/*ρ*)water (cm^2^/g)	(*µ*_en_/*ρ*)water (cm^2^/g)
Te K_α2_ x ray	27.202	0.406	0.415	0.207	0.399	0.210
Te K_α1_ x ray	27.472	0.757	0.408	0.201	0.390	0.203
Te K_β1,3,5_ x ray	30.98	0.202	0.337	0.140	0.358	0.141
Te K_β2,4_ x ray	31.71	0.0439	0.326	0.130	0.346	0.132
γ	35.492	0.0668	0.282	0.0943	0.294	0.0956

	Average energy (keV)	Total photons per disintegration		*Γ*_10keV_ (m^2^µGy/h/Bq)		

	28.37	1.476		0.0355		

^103^Pd Decays by electron capture *T*_1/2_ = 16.991 ± 0.019 d Limiting specific activity = 2.76 × 10^15^ Bq/g (7.47 × 10^4^ Ci/g)						
	Energy (keV)	Photons per disintegration	(*µ*/*ρ*)_air_ (cm^2^/g)	(*µ*_tr_/*ρ*)_air_ (cm^2^/g)	(*µ*/*ρ*)water (cm^2^/g)	(*µ*_en_/*ρ*)water (cm^2^/g)
Rh K_α2_ x ray	20.074	0.224	0.771	0.533	0.803	0.544
Rh K_α1_ x ray	20.216	0.423	0.759	0.521	0.790	0.532
Rh K_β1,3,5_ x ray	22.72	0 104	0.587	0.361	0.604	0.367
Rh K_β2,4_ x ray	23.18	0.0194	0.563	0.339	0.577	0.345
γ	39.75	0.00068	0.250	0.0695	0.249	0.0706
γ	357.5	0.00022	0.0998	0.0293	0.111	0.0325
γ	497.1	0.00004	0.0873	0.0297	0.0973	0.0330

	Average energy (keV)	Total photons per disintegration		*Γ*_10keV_ (m^2^µGy/h/Bq)		

	20.74	0.771		0.0361		

**Table 6a:** Correction factors for measurements made with the original WAFAC, assuming a source-to-aperture distance of 30 cm

Correction factor		For:	Currently implemented values		Values from the analyses presented in the text					
			^125^I	^103^Pd	^125^I	^125^I + 0.053 Ag Kx	^125^I + 0.094 Ag Kx	^125^I 0.195 Ag Kx	^125^I + 0.181 Pd Kx	^103^Pd
1	*k*_decay_	Correction to reference date, *T*_1/2_(d)	59.43	16.991	59.40	59.40	59.40	59.40	59.40	16.991
2	*k*_sat_	Recombination inside WAFAC	<1.004	<1.004	<1.004	<1.004	<1.004	<1.004	<1.004	<1.004
3	*k*_foil_	Attenuation in filter	1.0295	1.0738	1.0320	1.0342	1.0358	1.0394	1.0417	1.0776
4	*k*_att-WAFAC_	Aperture-to-WAFAC air attenuation	1.0042	1.0079	1.0051	1.0053	1.0054	1.0058	1.0060	1.0094
5	*k*_att-SA_	Source-to-aperture air attenuation	1.0125	1.0240	1.0143	1.0149	1.0153	1.0163	1.0170	1.0267
6	*k*_invsq_	Inverse-square correction for aperture	1.0089	1.0089	1.0089	1.0089	1.0089	1.0089	1.0089	1.0089
7	*k*_humidity_	Humidity correction	0.9982	0.9981	0.9979	0.9979	0.9979	0.9979	0.9979	0.9979
8	*k*_int-scatt_	In-chamber photon-scatter correction	0.9966	0.9962	0.9968	0.9968	0.9968	0.9967	0.9967	0.9964
9	*k*_stem_	Source-holder stem-scatter correction	0.9985	0.9985	0.9985	0.9985	0.9985	0.9985	0.9985	0.9985
10	*k*_elec_	In-chamber electron-loss correction	1.0	1.0	1.0	1.0	1.0	1.0	1.0	1.0
11	*k*_pen_	Aperture penetration	0.9999	0.9999	0.9999	0.9999	0.9999	0.9999	0.9999	0.9997
12	*k*_ext-scatt_	External photon-scatter correction	1.0	1.0	0.9947	0.9947	0.9947	0.9947	0.9947	0.9945

	Π *k*_3-12_		1.0489	1.1100	1.0486	1.0516	1.0538	1.0587	1.0621	1.1121

		Percent change			−0.03	+0.26	+0.47	+0.93	+1.26	+0.19

**Table 6b:** Correction factors for measurements made with the automated WAFAC, assuming a source-to-aperture distance of 30 cm

Correction factor		For:	Currently implemented values		Values from the analyses presented in the text					
			^125^I	^103^Pd	^125^I	^125^I + 0.053 Ag Kx	^125^I + 0.094 Ag Kx	^125^I + 0.195 Ag Kx	^125^I + 0.181 Pd Kx	^103^Pd
1	*k*_decay_	Correction to reference date, *T*_1/2_(d)	59.43	16.991	59.40	59.40	59.40	59.40	59.40	16.991
2	*k*_sat_	Recombination inside WAFAC	<1.004	<1.004	<1.004	<1.004	<1.004	<1.004	<1.004	<1.004
3	*k*_foil_	Attenuation in filter	1.0295	1.0738	1.0320	1.0342	1.0358	1.0394	1.0417	1.0776
4	*k*_att-WAFAC_	Aperture-to-WAFAC air attenuation	1.0042	1.0079	1.0048	1.0050	1.0051	1.0055	1.0057	1.0089
5	*k*_att-SA_	Source-to-aperture air attenuation	1.0125	1.0240	1.0143	1.0149	1.0153	1.0163	1.0170	1.0267
6	*k*_invsq_	Inverse-square correction for aperture	1.0089	1.0089	1.0089	1.0089	1.0089	1.0089	1.0089	1.0089
7	*k*_humidity_	Humidity correction	0.9982	0.9981	0.9979	0.9979	0.9979	0.9979	0.9979	0.9979
8	*k*_int-scatt_	In-chamber photon-scatter correction	0.9966	0.9962	0.9968	0.9968	0.9968	0.9967	0.9967	0.9964
9	*k*_stem_	Source-holder stem-scatter correction	0.9985	0.9985	0.9985	0.9985	0.9985	0.9985	0.9985	0.9985
10	*k*_elec_	In-chamber electron-loss correction	1.0	1.0	1.0	1.0	1.0	1.0	1.0	1.0
11	*k*_pen_	Aperture penetration	0.9999	0.9999	0.9999	0.9999	0.9999	0.9999	0.9999	0.9997
12	*k*_ext-scatt_	External photon-scatter correction	1.0	1.0	0.9947	0.9947	0.9947	0.9947	0.9947	0.9945

	Π *k*_3–12_		1.0489	1.1100	1.0483	1.0513	1.0535	1.0585	1.0618	1.1116

		Percent change			−0.06	+0.23	+0.44	+0.92	+1.23	+0.14

**Table 7:** Estimated relative standard uncertainties in the determination of air-kerma strength from prostate seeds using the WAFAC

Component	For:	Relative standard uncertainty, %		
		Type A	^125^I	^103^Pd

			Type B	Type B^b^
*I*_net,diff_	Net current	*s_I_*^a^	0.06	0.06
*W*/*e*	Mean energy per ion pair		0.15	0.15
*ρ*_0_	Air density		0.03	0.03
*d*	Source-aperture distance		0.24	0.24
*V*_eff_	Effective volume	0.11	0.01	0.01
*k*_decay_	Correction to reference date, *T*_1/2_(d)		0.02^b^	0.08^b^
*k*_sat_	Recombination inside WAFAC		0.05	0.05
*k*_foil_	Attenuation in filter		0.61	0.51
*k*_att-WAFAC_	Aperture-to-WAFAC air attenuation		0.08	0.10
*k*_att-SA_	Source-to-aperture air attenuation		0.24	0.31
*k*_invsq_	Inverse-square correction for aperture		0.01	0.01
*k*_humidity_	Humidity correction		0.07	0.07
*k*_int-scatt_	In-chamber photon scatter correction		0.07	0.07
*k*_stem_	Source-holder stem-scatter correction		0.05	0.05
*k*_elec_	In-chamber electron-loss correction		0.05	0.05
*k*_pen_	Aperture penetration		0.02	0.08
*k*_ext-scatt_	External photon scatter correction		0.17	0.19

Combined			0.754	0.719

a Determined as the standard deviation of the mean of the net current.

b Assuming time from the reference date is no more than ≈15 days.

**Table 8:** Ratio of NIST WAFAC standard for air-kerma strength to that of previous NBS standard (Loftus, 1984)

Model	# of seeds	Ratio (±2σ)
6702	6	0.898±0.014
6711	4	0.896±0.010
Both	10	0.897±0.011

## Footnotes

1 Due to the internal structure of the source, there might not be a practical calibration distance such that it behaves as a true point source.

2 A total of up to 4 coated spheres can be incorporated, for higher strength seeds.

3 Certain commercial equipment, instruments, or materials are identified in this paper to foster understanding. Such identification does not imply recommendation or endorsement by the National Institute of Standards and Technology, nor does it imply that the materials or equipment identified are necessarily the best available for the purpose.

4 Additional collector plates of 1 cm and 3 cm lengths, shown in Fig. 2, are not used in routine measurements.

5 Mylar is polyethylene terephthalate (PET), a product of DuPont.

6 An earlier design held the seed horizontally within several thin nylon monofilaments stretched between disks that were rotated; this was abandoned as seed mounting was far too tedious, less reproducible, and introduced some partial shielding that was difficult to characterize.

7 Charge is collected for an integral number of complete rotations (360°) for these measurements. The anisotropy is checked by measurements at fixed source orientations, typically, every 45°. The non-uniformity of air-kerma strength measured in the plane perpendicular to the seed axis can be significant, amounting to ±15 % or more, depending on seed construction.

8 The composition is mass fraction 0.89 W, 0.04 Cu and 0.07 Ni, with a density of 17.1 g/cm^3^.

9 This is accomplished by a layer of four small pieces of common office-quality double-stick transparent tape, whose spongy quality allows the fixing of seeds even with somewhat rounded end-caps. This much improved source-mounting arrangement was suggested by our colleague Christopher Soares (private communication).

10 Micarta is a brand name for a lightweight polymer.

11 The 4 mm W-alloy aperture plate is surrounded by 6 mm to 9 mm of brass, backed by 3.2 mm of Pb, which ensures that the penetration of photons outside the aperture is negligible except for the very-low-intensity high-energy (>240 keV) gamma rays emitted in ^103^Pd decay.

12 The very small component of scattered photons entering the aperture is treated separately, and their contribution to the penetration of the aperture edge is negligible.

13 There are, in principle, also some internally scattered photons emerging from the source, but these continuum photons are negligible in number compared to the strongly dominant monoenergetic photons.

14 For the short collecting lengths, the field lines are all nearly parallel with no significant bulging.

15 The equation appears to be in essential agreement with the work of Jones [36,37].

16 Following current conventions, uncertainties are classified into two categories: Type A includes those evaluated by statistical methods, and Type B includes those evaluated by other means (usually scientific judgment).
