# Erratum: New National Air-Kerma-Strength Standards for ^125^I and ^103^Pd Brachytherapy Seeds

**DOI:** 10.6028/jres.109.021

**Published:** 2004-04-01

**Authors:** Stephen M. Seltzer, Paul J. Lamperti, Robert Loevinger, Michael G. Mitch, James T. Weaver, Bert M. Coursey

**Affiliations:** National Institute of Standards and Technology, Gaithersburg, MD 20899-0001.

[J. Res. Natl. Inst. Stand. Technol. Volume 108, Number 5, September–October 2003, p. 337]

On page 351, in Table 5, the units of *Γ*_10keV_ for both ^125^I and ^103^Pd should be “m^2^μGy h^−1^ MBq^−1^.”

